# Molecular characterization and ligand binding specificity of the PDZ domain-containing protein GIPC3 from *Schistosoma japonicum*

**DOI:** 10.1186/1756-3305-5-227

**Published:** 2012-10-10

**Authors:** Yi Mu, Shuai Liu, Pengfei Cai, Youhe Gao

**Affiliations:** 1 National Key Laboratory of Medical Molecular Biology, Dept. of Physiology and Pathophysiology, School of Basic Medicine, Peking Union Medical College, Institute of Basic Medical Sciences, Chinese Academy of Medical Sciences, Beijing 100005, P.R. China; 2 MOH Key Laboratory of Systems Biology of Pathogens, Institute of Pathogen Biology, Chinese Academy of Medical Sciences & Peking Union Medical College, Beijing 100730, P.R. China; 3 Donnelly Centre for Cellular and Biomolecular Research, University of Toronto, Toronto, ON, Canada

**Keywords:** *Schistosoma japonicum*, GIPC3, PDZ domain-containing protein, Ligand binding specificity

## Abstract

**Background:**

Schistosomiasis is a serious global health problem that afflicts more than 230 million people in 77 countries. Long-term mass treatments with the only available drug, praziquantel, have caused growing concerns about drug resistance. PSD-95/Dlg/ZO-1 (PDZ) domain-containing proteins are recognized as potential targets for the next generation of drug development. However, the PDZ domain-containing protein family in parasites has largely been unexplored.

**Methods:**

We present the molecular characteristics of a PDZ domain-containing protein, GIPC3, from *Schistosoma japonicum* (SjGIPC3) according to bioinformatics analysis and experimental approaches. The ligand binding specificity of the PDZ domain of SjGIPC3 was confirmed by screening an arbitrary peptide library in yeast two-hybrid (Y2H) assays. The native ligand candidates were predicted by Tailfit software based on the C-terminal binding specificity, and further validated by Y2H assays.

**Results:**

SjGIPC3 is a single PDZ domain-containing protein comprised of 328 amino acid residues. Structural prediction revealed that a conserved PDZ domain was presented in the middle region of the protein. Phylogenetic analysis revealed that SjGIPC3 and other trematode orthologues clustered into a well-defined cluster but were distinguishable from those of other phyla. Transcriptional analysis by quantitative RT-PCR revealed that the *SjGIPC3* gene was relatively highly expressed in the stages within the host, especially in male adult worms. By using Y2H assays to screen an arbitrary peptide library, we confirmed the C-terminal binding specificity of the SjGIPC3-PDZ domain, which could be deduced as a consensus sequence, -[SDEC]-[STIL]-[HSNQDE]-[VIL]*. Furthermore, six proteins were predicted to be native ligand candidates of SjGIPC3 based on the C-terminal binding properties and other biological information; four of these were confirmed to be potential ligands using the Y2H system.

**Conclusions:**

In this study, we first characterized a PDZ domain-containing protein GIPC3 in *S. japonicum*. The SjGIPC3-PDZ domain is able to bind both type I and II ligand C-terminal motifs. The identification of native ligand will help reveal the potential biological function of SjGIPC3. These data will facilitate the identification of novel drug targets against *S. japonicum* infections.

## Background

Schistosomiasis, caused by the parasitic blood fluke of the genus *Schistosoma*, remains a major global public health problem that affects more than 230 million people living in the endemic areas of 77 countries worldwide (more than 95% in Africa) (http://www.who.int/mediacentre/factsheets/fs115/en/index.html). Three major pathogenic schistosome species, *Schistosoma mansoni*, *Schistosoma haematobium*, and *Schistosoma japonicum*, are known to contribute to the disease loads. The treatment of schistosomiasis is based on the long-term mass treatment with the only available drug, praziquantel (PZQ). However, concerns about the problem of drug resistance have necessitated a search for alternative treatments [1-3]. The decoding of the genomes of three major pathogenic blood flukes has provided a valuable entity for the systematic identification of possible drug targets against the parasite [4-6].

Protein-protein interactions (PPIs) offer a realm of less explored potential drug targets, and are recognized to be the novel targets of next-generation drug development [7]. The PDZ domain is one of the most important PPI modules, and is emergently regarded as a novel target of drug discovery [8,9]. PDZ domains are built of approximately 70 to 90 residues and form a compact globular fold consisting of a core of six β-strands (βA - βF) and two α-helices (αA, αB) [10]. The C-terminus (4 to 5 C-terminal residues) of partners usually projects to a hydrophobic pocket formed with the carboxylate-binding loop (βA-βB loop), which contains the conserved “GLGF” motif, and the αB helix of the PDZ domain [11]. The PDZ binding motifs have been conventionally placed into four classes based on the extreme C-terminus: Class I, -[S/T]-XΦ*; Class II, -ΦXΦ*; Class III, -[D/E/K/R]-XΦ*; and Class IV, -*Xψ*-[D/E]*; where *X* is any amino acid, *Φ* is a hydrophobic residue, *ψ* is an aromatic residue, and * represents the stop codon of the peptide [12]. Although the PDZ domains are conserved in a variety of organisms, no detailed literature about the schistosome-derived PDZ domain has been reported thus far.

As a benefit of the decoding of the genome, several PDZ domain-containing proteins were deposited in the public *S. japonicum* database. Among them, one is homologous to the mammalian GAIP-interacting proteins, C terminus (GIPCs, also known as TIP-2, GLUT1CBP, RGS19IP1, SEMCAP-1, and synectin); here we have designated the *S. japonicum* homologue as SjGIPC3. GIPCs are characterized by a single, centrally located PDZ domain for ligand binding. GIPC1 was originally found to bind to the G_αi_ GTPase-activating protein RGS-GAIP and have been suggested to exert function in a G-protein coupled complex mediating vesicular trafficking [13]. Accumulating data have confirmed that the PDZ domain of GIPCs can interact with a panel of partners through C-terminal region recognition. Most of these partners are transmembrane receptors, such as Tax [14], M-semF [15], Glut1 [16], neuropilin-1 [17], Syndecan-4 [18], and so on [19-28]. In addition, human and *Drosophila* GIPC orthologues can also bind to the non-receptor protein, myosin VI [29,30]. Recently, GIPC orthologues were regarded as novel and potential targets for therapeutic development for the treatment of human cancers [31,32]. Several cell-permeable octapeptides targeting the PDZ domain of GIPCs were designed based on binding specificity and have shown their powers in inhibition of proliferation and induction of apoptosis in some types of cancer [31,33].

In this study, we present for the first time the molecular characteristics of a PDZ-containing protein from *S. japonicum*, GIPC3. We analyzed the expression levels of *SjGIPC3* during development of the parasite. To reveal its ligand binding specificity, a random peptide library constructed from human genomic DNA was used in yeast two-hybrid (Y2H) assays to screen for the C-terminal binding motifs of the SjGIPC3-PDZ domain. Furthermore, the potential ligands of SjGIPC3 were predicted by bioinformatic approaches based on the C-terminal binding specificity, and validated in the Y2H system. This work will facilitate the identification of novel drug targets against *S. japonicum*.

## Methods

### Parasites and animals

The *S. japonicum*-infected *Oncomelania hupensis* were purchased from Jiangxi Institute of Parasitic Diseases, Nanchang, China. Cercariae were shed from the infected snails and harvested for total RNA isolation. New Zealand White rabbits were percutaneously infected with cercariae. Hepatic schistosomula and adult worms were obtained from the rabbits by hepatic-portal perfusion at 2 and 6 weeks post-infection, respectively. Male and female adult worms were manually separated with the aid of a light microscope. Eggs were isolated from liver tissues of rabbits after 6 weeks of infection by the sieving and enzymatic digestion method [34].

### Bioinformatics analysis of SjGIPC3

The nucleotide and deduced amino acid sequence of SjGIPC3 were analyzed from NCBI. The PDZ domain of SjGIPC3 protein was predicted in the Conserved Domain Database (CDD) v.2.25 of NCBI (http://www.ncbi.nlm.nih.gov/cdd) [35]. The three-dimensional structure of the SjGIPC3-PDZ domain was predicted by the Rosetta server (http://robetta.bakerlab.org/) [36] with the PDZ domain in GIPC2 (PDB code: 3GGE – chain A) as the template. The 3D structure was further refined with PyMOL Viewer program (http://www.pymol.org/).

### Multiple sequence alignment and phylogenetic analysis

The GIPC homologous sequences of other species were retrieved from GenBank using SjGIPC3 as bait. Protein sequences were aligned by ClustalX 2.0. The alignment was refined with GeneDoc software. Phylogenetic analysis was performed as previously described [37]. The alignment was further optimized by deletion of highly variable N- and C-terminal regions. The phylogenetic tree was constructed with MEGA 4 using the neighbor-joining method. Bootstrap values were expressed as percentage of 1000 replicates.

### Quantitative RT-PCR

Total RNAs of *S. japonicum* at different developmental stages (cercariae, hepatic schistosomula, separated adult male and female worms, and eggs) were extracted using Trizol reagent (Invitrogen). The contaminating genomic DNA was removed from RNA samples with TURBO DNA-free^TM^ kit (Ambion, CA, USA). RNA quantification and quality control was conducted by 1% agarose gel electrophoresis and Nanodrop ND-1000 spectrophotometer (Nanodrop Technologies, Wilmington, DE). For each sample, 1 μg total RNA was reverse transcribed into first-strand cDNA using SuperScript^TM^ III Reverse Transcriptase Kit (Invitrogen), with Oligo dT (15) primer. cDNA synthesis was performed as follows: 25°C for 5 min, 50°C for 1 h, 70°C for 15 min. Each PCR reaction contained 12.5 μl of 2×Brilliant II SYBR Green QPCR Master Mix (Agilent, USA), 1 μl diluted cDNA (20×), 1 μl of the forward and reverse primer pair (final concentration: 0.2 μM each primer, Additional file 1 Table S1), and 10.5 μl of sterile water. The PCR program included 40 cycles with denaturation at 95°C for 30 s, followed by annealing and extension at 60°C for 1 min. Quantification of the relative differential expression for *SjGIPC3* gene was performed by normalizing against the *PSMD4* transcript (26S proteasome non-ATPase regulatory subunit 4, GenBank accession number: FN320595) and applying the comparative 2^-ΔΔCt^ method, according to the SDS 1.4 software (Applied Biosystems, Foster City, USA) [38].

### Construction of bait plasmids

The DNA fragments encoding full-length SjGIPC3 and the PDZ domain region (AA 99–195) were amplified from the adult worm cDNA templates using high fidelity Phusion DNA polymerase (Finnzymes Oy, Finland) (Primer sets are listed in Additional file 1 Table S1). The PCR was performed with an initial denaturation for 1 min at 98°C. Thirty PCR cycles were performed as follows: 98°C for 5 s, 50°C for 30 s, and 72°C for 15 s. The final extension was 5 min at 72°C. The amplicons were digested with *Eco*RI and *Bam*HI, and cloned into GAL4 BD vector, pBridge. The recombinant plasmids were transformed into DH5α (DE3) *Escherichia coli* and positive clones were selected for sequencing.

### Yeast two-hybrid screening of random peptide library

Each GAL4 BD-fusion bait plasmid was transformed into the yeast strain CG1945 using the lithium acetate procedure. The transformants were grown on SD/-Trp plates and lacZ assays were performed to examine self-activation. The transformants were further spread on SD/-His/-Trp plates with different concentration of 3-amino-1,2,4-triazole for leakage test. The random peptide library (constructed with *Tsp*509I-digested human genomic DNA fragments) [39] was screened following the MATCHMAKER Two-Hybrid System protocol (Clontech). The transformants were screened on SD/-Trp/-His/-Leu plates with different concentrations of 3-amino-1,2,4-triazole (2.5 mM for full-length SjGIPC3 bait transformants, and 20 mM for SjGIPC3-PDZ bait transformants) and further verified by the improved LacZ assays. After rescue, the potential positive plasmids were isolated and retransformed into the yeast strain CG1945 containing corresponding bait plasmid. Only the clones that were positive for all the reporter assays and confirmed by at least two independent tests were selected for specific interactions and sequenced [40].

### Prediction of candidate ligands

A consensus C-terminal binding sequence of SjGIPC3-PDZ domain was deduced from sequence alignment of positive clones from yeast two-hybrid screening. The *S. japonicum* predicted protein sequences were downloaded from SDSPB (http://lifecenter.sgst.cn/schistosoma/cn/schdownload.do). The Tailfit software [41] was used to search against the *S. japonicum* protein sequences to retrieve potential ligand proteins whose carboxyl termini matched the consensus-binding sequence. These peptide sequences were then manually blasted against NCBI's non-redundant protein database to filter the truncated fragments lacking of the C-terminus. The most promising candidate ligand proteins were selected further based on biological information such as subcellular localization and potential molecular function.

### Confirmation of candidate SjGIPC3-ligand interactions

The GAL4 AD plasmids expressing the carboxyl-terminus of the candidate ligands of the SjGIPC3-PDZ domain were first constructed. The self primer template PCR was performed to produce DNA fragments encoding the 10–11 amino acid residues of extreme carboxyl-terminus of potential ligands (The primer sets were shown in Additional file 1 Table S1). The PCR procedure included thirty cycles as follows: 98°C for 5 s, 50°C for 25 s, and 72°C for 1 s, and a final extension at 72°C for 2 min. The resulting DNA fragments were digested with *Eco*RI and *Bam*HI endonucleases, and cloned into the *Eco*RI/*Bam*HI sites of GAL4 AD vector, pGADT7 followed by sequencing. Each constructed plasmid was co-transformed with the *SjGIPC3*-PDZ domain or full-length *SjGIPC3* bait plasmid into yeast strain CG1945, respectively. The positive interactions were selected in the yeast two-hybrid system as mentioned above.

## Results

### Molecular characteristics of *S. japonicum* GIPC3

**Figure 1:**
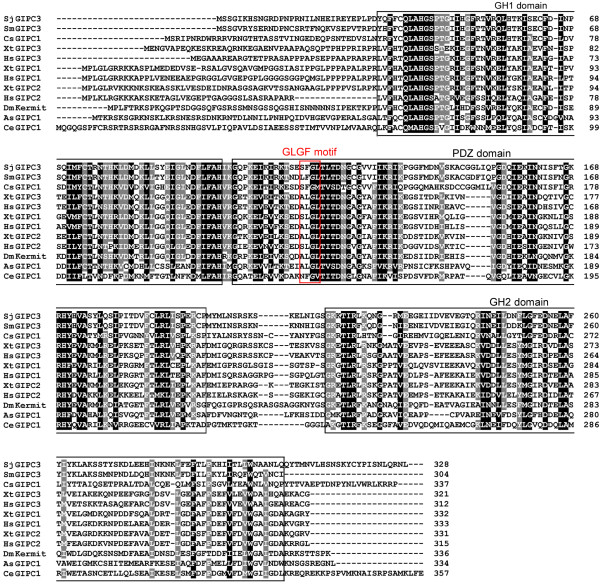
**Sequence alignment of peptide sequences of SjGIPC3 and GIPC orthologues derived from other species with ClustalX 2.0. **Black indicates identity in all variants; grey indicates a conservative substitution in at least one variant; white indicates a non-conservative residue; dashes indicate missing residues. GenBank Accession Numbers: SjGIPC3, *Schistosoma japonicum*, (AAW24514.1); SmGIPC3, *Schistosoma mansoni*, (XP_002569948); CsGIPC1, *Clonorchis sinensis*, (GAA51139); XtGIPC3, *Xenopus tropicalis*, (NP_001096502); HsGIPC3, *Homo sapiens*, (NP_573568); XtGIPC1, *Xenopus tropicalis*, (NP_001011331); HsGIPC1, *Homo sapiens*, (NP_005707); XtGIPC2, *Xenopus tropicalis*, (NP_001120269); HsGIPC2, *Homo sapiens*, (NP_060125); DmKermit, *Drosophila melanogaster*, (NP_652028); AsGIPC1, *Ascaris suum*, (ADY44129); CeGIPC1, *Caenorhabditis elegans*, (NP_498017). The GH1, GH2, and PDZ domains are marked with black boxes and the boundaries for these domains were defined based on the previous study [28]. The “GLGF” motif region within the PDZ domains of GIPC orthologues is highlighted by the red box.

**Figure 2:**
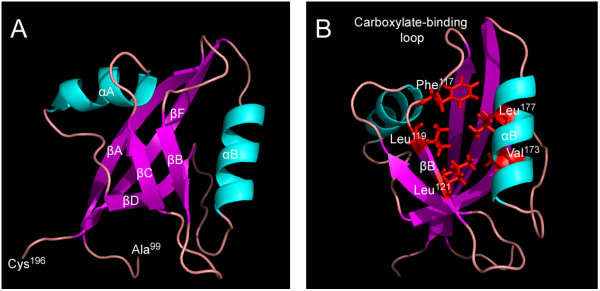
**The cartoon diagrams for the 3D structure of the SjGIPC3-PDZ domain. A**. The SjGIPC3-PDZ domain is colored by secondary-structure elements (α-helices, cyan; β-strands, magenta; loops, salmon). **B**. A hydrophobic pocket was formed by the αB helix, the βB strand, and the carboxylate-binding loop. The hydrophobic amino acids within the pocket are highlighted in red. The residue **F **in the unique “GLGF” motif (here is “S**F**GL”) may affect the selection of the C-terminal tails of its partner proteins.

By searching the *S. japonicum* proteomic database, we found that at least 25 PDZ domains are distributed among 16 proteins. *S. japonicum* GIPC3 is a single PDZ domain-containing protein, which is 328 amino acids in length, with a theoretical molecular weight of 37 kDa. By performing a CD-search, we found that the PDZ domain was located in the middle region of SjGIPC3 protein. The peptide sequence of SjGIPC3 was aligned with sequences of orthologues from other taxa. The GIPC homology 1 (GH1) and PDZ domains of GIPC proteins were relatively well conserved, in contrast to the GIPC homology 2 (GH2) domain and the flanking N- and C-terminal regions (Figure 1). Homology analysis revealed that SjGIPC3 shares a relatively high degree of sequence homology with *S. mansoni* orthologue (GenBank Accession number: XP_002569948, identity=78%, only aa1894-2197 region was used for calculating identity) and followed by share 46% identity with *Clonorchis sinensis* orthologue (GenBank Accession number: GAA51139). The sequence in the conserved “GLGF” motif is replaced by “SFGL” in SjGIPC3, which is different to that in any other GIPC orthologues (Figure 1). Structural prediction revealed that the SjGIPC3-PDZ domain was comprised of two α-helixes and five β-sheets (lacking βE, Figure 2A), with a hydrophobic pocket formed between βB and αB, in which the residue **F** from the unique “GLGF” motif (“S**F**GL” in the SjGIPC3-PDZ domain, while “ALGL” or “SLGL” in the PDZ domains of mammalian GIPCs) may affect the ligand binding specificity of the PDZ domain (Figure 2B).

### Phylogenetic analysis of SjGIPC3

**Figure 3:**
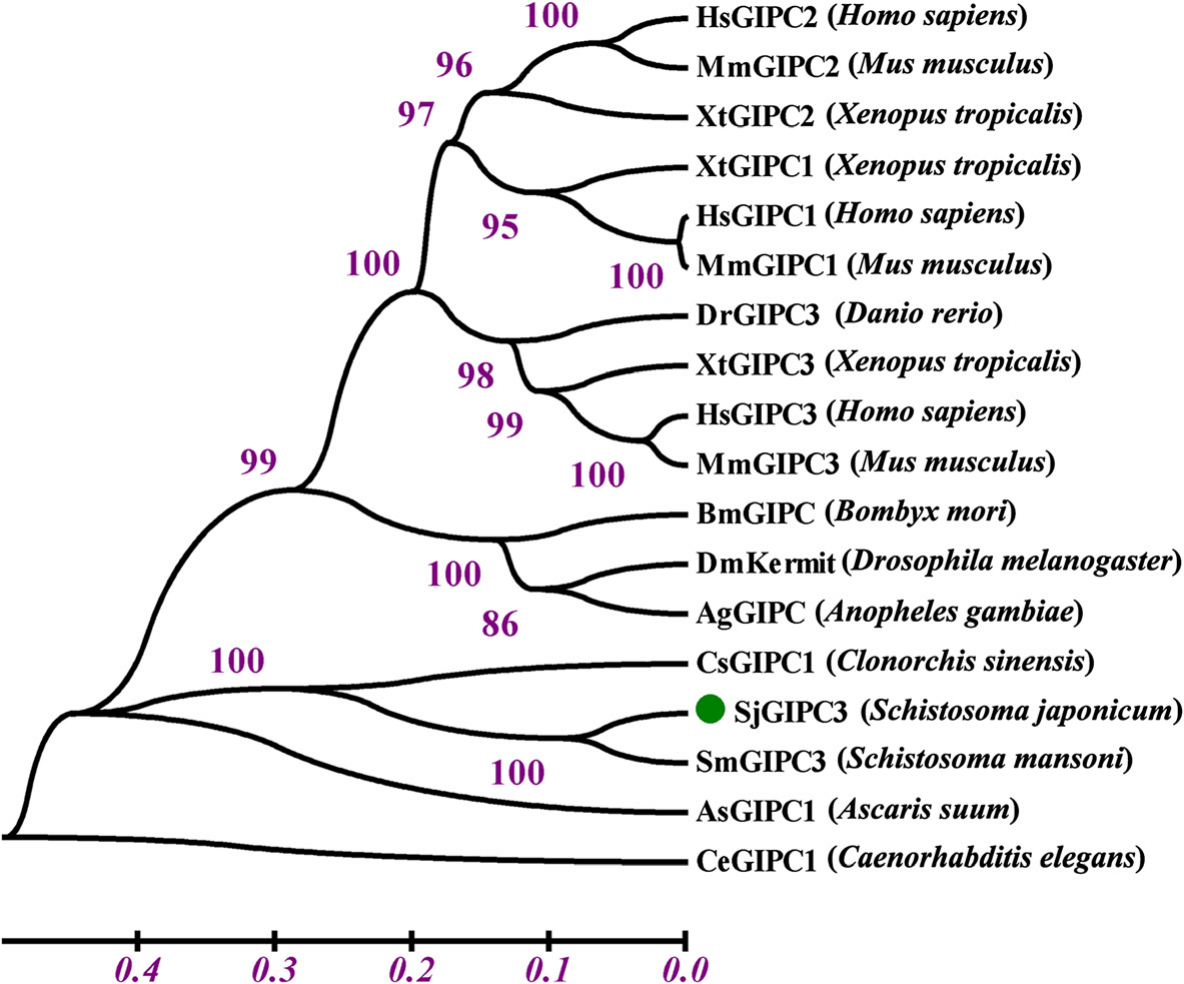
**Phylogenetic tree of SjGIPC3 and its orthologues derived from other species. **Bootstrap percentages are indicated at each branch. GenBank Accession Numbers: HsGIPC2 (NP_060125), MmGIPC2 (NP_058563), XtGIPC2 (NP_001120269), XtGIPC1 (NP_001011331), HsGIPC1 (NP_005707), MmGIPC1 (NP_061241), DrGIPC3 (CAX13825), XtGIPC3 (NP_001096502), HsGIPC3 (NP_573568), MmGIPC3 (NP_683753), BmGIPC (NP_001040127), DmKermit (NP_652028), AgGIPC (XP_308197) CsGIPC1 (GAA51139), SjGIPC3 (AAW24514.1), SmGIPC3 (XP_002569948), AsGIPC1 (ADY44129), CeGIPC1 (NP_498017).

The sequences of GIPC orthologues from other taxa were retrieved to create a phylogenetic tree using the Neigh-bor-Joining method. SjGIPC3 was clustered with *S. mansoni* and *C. sinensis* orthologues to form a clade, which was divergent from those of other phyla, even with the parasitic nematode-derived orthologue, AsGIPC1 (Figure 3). However, less is known about whether this divergence of fluke GIPC orthologues is related to adaptations specific to their parasitic life.

### Transcriptional analysis of the *SjGIPC3* gene at different developmental stages of the parasite

**Figure 4:**
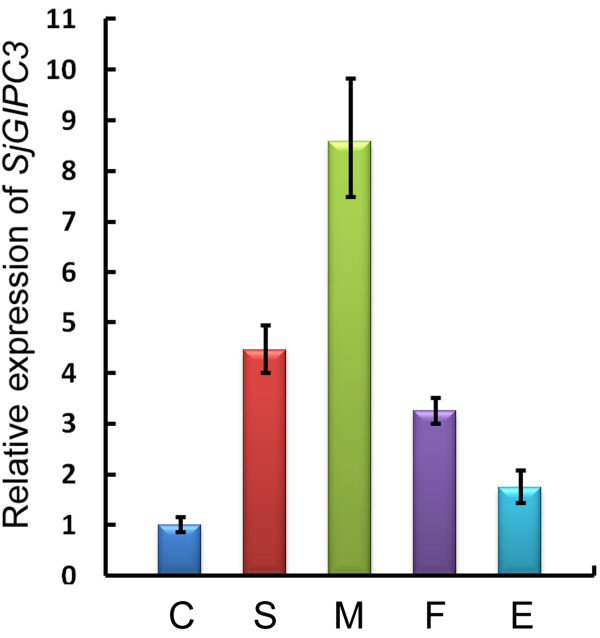
**Determination of relative expression levels of *SjGIPC3 *transcripts at different developmental stages by qRT-PCR. **Transcriptional levels were calibrated according to the comparative 2^−ΔΔCt^ method using the housekeeping gene *SjPSMD4* as an endogenous control, and were normalized relative to the cercarial stage. Error bars represent the standard deviations of the mean from the three replicates. C, cercariae; S, hepatic schistosomula; M, male adult worms; F, female adult worms; E, eggs.

To determine the transcriptional patterns of *SjGIPC3* at different developmental stages and between sexes, qRT-PCR was performed using an optimal reference gene, 26S proteasome non-ATPase regulatory subunit 4 (*PSMD4*), as an internal control [42]. As a result, we observed that the *SjGIPC3* gene was ubiquitously expressed at different developmental stages, but in a stage-biased pattern. The expression level of the *SjGIPC3* gene was lower in the cercarial stage than in any other stages in which it was detected, suggesting that the function of SjGIPC3 is mainly exerted in the stages within the host. In adult worms, the transcriptional level of *SjGIPC3* was significantly higher in male than in female worms (Figure 4).

### Determination of the ligand binding specificity of SjGIPC3

To investigate the binding properties of the PDZ domain of SjGIPC3, we screened an arbitrary peptide library using Y2H assays. When the library was screened against the SjGIPC3-PDZ domain, 37 positive clones were sequenced, each encoding a unique carboxy-terminus. Among them, 17 belong to Class I PDZ binding motifs, 16 belong to Class II PDZ binding motifs, and four are unclassified (Table 1). When the library was screened against the full-length SjGIPC3, 42 positive clones were obtained. Sequence analysis revealed that three pairs of clones were identical; thus a total of 39 unique carboxy-terminal sequences were obtained. Among them, 24 belong to Class I PDZ binding motifs, 12 belong to Class II PDZ binding motifs, and three are unclassified (Table 1).

**Table 1:** The unique ligand carboxyl termini obtained from Y2H assays using the SjGIPC3-PDZ domain or full-length SjGIPC3 as bait

**The SjGIPC3-PDZ domain as bait**			**Full-length SjGIPC3 as bait**		
**Class I**	**Class II**	**Unclassified**	**Class I**	**Class II**	**Unclassified**
-YTEGETSL*	-TCTLELKI*	-FMVMDSQC*	-LFYKDSEI*	-VFLDEISI*	-TKLSESQC*
-YPLFSSHL*	-KLGNFLDI*	-CCWMECSV*	-YLYSCSEI*	-GNSTEINI*	-KDSQESHC*
-KGKECSSI*	-KLGNSIDV*	-PLASDCEV*	-KLGNCSHI*	-DNLEDIEI*	-ALWLDLDC*
-AGHSGSCL*	-ESPFYLNI*	-LIITINLY*	-CSWLCSDV*	-ACDSEVSL*	
-FRYQQTDI*	-VIKLEINL*		-LSASESEV*	-VMNNTVEF*	
-DFCKKSEI*	-LHTNDISV*		-REVTCSEI*	-PEPWCITL*	
-GNSSFSVL*	-RWENYIEI*		-LWLGESNV*	-FKKLEIDL*	
-DSFSTTTL*	-PEAGELLL*		-KLGNCSEI*	-DRYRDIEI*	
-IGNSDTQL*	-NMSDNLDL*		-RNHVESNI*	-TLNEDIEF*	
-STISMSSI*	-LGNYDISL*		-VSFSETTL*	-FLICEIEI*	
-GATDASHI*	-YYSSEVQI*		-LLLNETNI*	-EWPLSISF*	
-FCKVDSDV*	-GNFSEINI*		-LGNFETEV*	-NWQLDLEV*	
-SDFYSSVL*	-LLENSINL*		-ICFYSSEI*		
-LCGNRSQV*	-LSFLCIEI*		-KEQQESDL*		
-DICWESYV*	-HCSEDLIF*		-FSPRETNL*		
-ARCGSSCL*	-EEESELEM*		-NSDKPSHT*		
-LTQSHSML*			-QQHNESEL*		
			-HNSNESSF*		
			-GRINESAI*		
			-LGNLESHI*		
			-EGQGCSEL*		
			-KLGNYSEI*		
			-GASSVSEV*		
			-VFLDETHI*		

**Figure 5:**
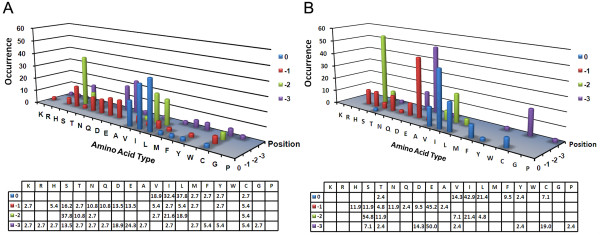
**Binding properties of the SjGIPC3-PDZ domain. **The four amino acids from the extreme carboxyl terminus of positive clones isolated from Y2H assays with the SjGIPC3-PDZ domain as bait (**A**), or full-length SjGIPC3 as bait (**B**), respectively, were aligned and the occurrence for each amino acid at each position was statistically analyzed. The positions were numbered from the carboxyl terminus (position 0). The tables described the percentage of each amino acid type at each given position (blank: percentage = 0%).

In the first assay using the SjGIPC3-PDZ domain as bait, the domain showed predominant preference for hydrophobic amino acids at the extreme carboxyl terminus (P^0^), especially Leu (37.8%), Ile (32.4%), or Val (18.9%). The P^-1^ of the carboxyl terminus slightly prioritizes polar amino acids, such as Ser (16.2%), Asn (10.8%), or Gln (10.8%), and acidic amino acids, such as Asp (13.5%) or Glu (13.5%). At P^-2^, the polar amino acids Ser (37.8%) and Thr (10.8%) and the aliphatic amino acids Ile (21.6%) and Leu (18.9%) were preferentially selected. At P^-3^, Ser (13.5%), Asp (18.9%), and Glu (24.3%) were preferred, along with the rare occurrence of other amino acids (Figure 5A). Based on the statistical analysis of unique carboxy-terminal sequences, a consensus-binding sequence can be deduced as -[S/D/E]-[S/T/I/L]-[S/N/Q/D/E]-[V/I/L]*.

In the second assay, full-length SjGIPC3 was probed to screen the random peptide library, and a more significant binding specificity emerged. As in the first assay, hydrophobic amino acids, especially Ile (42.5%), Leu (21.3%), or Val (14.2%), were predominantly preferred at P^0^, with a slight bias toward the aromatic amino acid Phe (9.5%). At P^-1^, the preference for Glu (45.2%) was dramatically increased when compared with the first assay, along with a rare selection of other residues: His (11.9), Ser (11.9%), Asn (11.9%), or Asp (9.5%). The P^-2^ position demonstrated a dramatic preference for the polar amino acid Ser (54.8%) and the hydrophobic amino acid Ile (21.4%); other amino acids, such as Thr (11.9%), Val (7.1%), or Leu (4.8%), also occurred at a low rate. At P^-3^, Glu (45.2%) was predominantly selected, followed by Cys (19.0%) and Asp (14.3%) (Figure 5B). Similar to the first assay, a consensus-binding sequence can be inferred from the second Y2H assay: -[D/E/C]-[S/T/I]-[H/S/N/D/E]-[V/I/L]*. Finally, based on both assays, the consensus-binding sequence of the SjGIPC3-PDZ domain could be reduced to -[SDEC]-[STIL]-[HSNQDE]-[VIL]*, which fits in both class I and class II binding motifs.

### Prediction and identification of native ligands of SjGIPC3

We searched for the potential native ligands of SjGIPC3 in the *S. japonicum* predicted proteomic database (sjr2_protein.fasta) with the Tailfit program using the consensus-binding sequence -[SDEC]-[STIL]-[HSNQDE]-[VIL]* as bait; 92 proteins that physically fit the consensus binding sequence were fished out. We also performed BLAST searching on NCBI within taxa 6182 directly; using each unique carboxy-terminal sequence (the extreme four amino acids) obtained from both Y2H assays as bait. A panel of proteins whose C-terminal tail matched the unique sequences was retrieved. The protein sequences retrieved from both bioinformatics pipelines were used to perform BLASTP searches on NCBI to manually filter those peptides lacking the C-terminus. We also used integrated biological information, such as transmembrane region prediction, potential molecular functions, and the C-terminal sequence consensus between *S. japonicum* and *S. mansoni* to determine the most promising candidate ligands. Finally, six proteins were selected as ligand candidates of SjGIPC3 (Table 2). Among them, four were further confirmed to be potential ligands of SjGIPC3 in the Y2H system.

**Table 2:** Validation of the predicted ligand candidates of SjGIPC3 in the Y2H system

**Bait Sequences**	**Code**	**ID**	**C-terminus (*S. japonicum*)**	**C-terminus (S*. mansoni*)**	**Protein Description**	**Results**^**3**^	**Results**^**4**^
C-terminal tails^1^	L1	ACE06921	-PDERTTTL*	-HNERTTTL*	glutamate receptor NMDA	√	√
	L2	AAX27986	-PNHLCSDV*	-SNYLCSDV*	hypothetical protein	√	√
-[SDEC]-[STIL]-[HSNQDE]-[VIL]*^2^	L3	CPRT0000005506	-DESNSLHV*	-EDGNSLSA*	tetraspanin (Sj25)	×	×
	L4	CPRT0000006203	-DDRLCSQV*	-DDRLCSQV*	hypothetical protein	√	√
	L5	CPRT0000007433	-ELLTSTEV*	-ELLTSTEV*	Asparagine-rich protein	√	√
	L6	CPRT0000008390	-KSNVSLSV*	-KSNVSLSV*	putative rhodopsin-like orphan GPCR	×	×

^1^: The extreme four amino acids of each unique ligand carboxyl terminus listed in Table 1 were used to BLAST on NCBI to retrieve potential ligands within taxa 6182 (*S. japonicum*).

^2^: The Tailfit software was used to search against the *S. japonicum* proteomic database to retrieve potential ligands with the consensus-binding sequence;

^3^: The validation results from Y2H assays, using the SjGIPC3-PDZ domain as bait.

^4^: The validation results from Y2H assays, using full-length SjGIPC3 as bait.

√: positive in the Y2H system; ×: negative in the Y2H system.

## Discussion

Domain loss events are extensively widespread in *S. japonicum*, which serves as a notable consequence of the adoption of parasitism [4]. It is estimated that 1,000 protein domains have been abandoned by *S. japonicum* during long-term evolution, which suggests that the remaining domains are necessary for parasitic growth, development, and maturation. The proteomic information revealed that at least 25 PDZ domains are distributed among 16 *S. japonicum* proteins, suggesting that the PDZ domain is one of the important modules reserved in the parasite to mediate multiple biological processes. In the present study, we have characterized one of the PDZ domain-containing proteins from *S. japonicum*, GIPC3, for the first time. The ligand binding properties of the SjGIPC3-PDZ domain were determined by Y2H assays, and its potential ligands were identified.

Screening a highly diverse peptide library provided an opportunity to fish out the potential binding property of SjGIPC3. Whether the SjGIPC3-PDZ domain or full-length SjGIPC3 were used as bait, analogous binding specificities can be deduced from the unique C-terminal tails obtained from both Y2H assays. However, the GH1 and GH2 domains of SjGIPC3 may still affect the binding specificity of the PDZ domain to some degree, because the ligand motifs were more canonical when full-length SjGIPC3 was used as bait. For instance, at P^-1^, the acidic amino acid Glu was more preferentially selected in the second assay (45.2%) than in the first assay (13.5%). Similarly, at P^-3^, 16 types of amino acids emerged in the first assay, while only eight kinds of amino acids were presented in the second assay. Also at this position, the occurrence of Glu was 50.0% when screened against the full-length SjGIPC3, twice the occurrence rate as when the SjGIPC3-PDZ domain was used as bait. Thus, the binding specificity screened using full-length SjGIPC3 as bait may be more authentic to its native status.

Previously, GIPCs have been shown to interact specifically with Class I (−*X*-S/T-XΦ*), II (−ΦXΦ*), and III (−*XX*-C*) binding motifs [43]. Moreover, Kermit, the GIPC orthologue in *Drosophila*, and GLUT1CBP each interact with Myosin VI through an internal sequence within the cargo binding domain [43,44]. In this study, we focused on the C-terminal binding specificity of SjGIPC3 and found that the PDZ domain of SjGIPC3 preferred to bind to Class I and II motifs, consistent with the extensive ligand-binding ability of GIPC orthologues in other species [43]. However, regarding the binding of Class II motifs, the only example presented thus far is that the PDZ domain of synectin binds to sydecan-4 through the “-EFYA*” motif, in which the aromatic amino acid Phe was selected at P^-2^[18]. In our study, it is worth noting that this site can be occupied by the aliphatic amino acid residues Ile and Leu, in addition to Ser and Thr (Table 1). Moreover, in a previous report, the binding motifs of GIPC orthologues trend toward selecting a Val, Ala, or Cys residue at p^0^[43]; here, the more hydrophobic amino acids, such as Ile and Leu, were predominantly preferred. These diverse binding characteristics could be utilized for drug development against SjGIPC3.

On average, one PDZ protein is capable of interacting with 17 partners [45]. Although a panel of *S. japonicum* proteins deposited in the public database bears a C-terminal tail fittting in the consensus binding sequence, the bioinformatic hints at molecular function and localization have prevented them from being ligand candidates of SjGIPC3. Using the Y2H assay, four proteins were determined to be potential ligand candidates for SjGIPC3, which suggests that some other potential ligands may not have been discovered yet, likely restricted by the quality of the proteomic database, as truncated fragments have been extensively deposited in the *S. japonicum* proteomic database.

To adapt to the complexity of the life cycle, schistosomes are equipped with a complex nervous system to sense environmental signals [46]. Transcriptional analysis has revealed that the expression of *SjGIPC3* was dramatically up-regulated after invasion of the host, suggesting that it may play an important role (e.g., signal transduction) in the context of the parasite-host interaction. More importantly, the preferential expression of *SjGIPC3* in male rather than female adult worms further hinted that the potential function of the protein was related to the processing of environmental information, as male adult worms interface with the host and female adult worms resided in the gynecophoral canal, more stimulating signal would be received by male adult worms [47]. GIPC3 mutations have recently been reported to cause autosomal recessive nonsyndromic sensorineural hearing loss [48-50]. Previously, Yi and his colleagues suggested that the GIPC orthologue in mouse might associate with extrasynaptic NMDA receptors through binding to the C-terminal tail “ESDV”, and may be involved in the organization and trafficking of this population of receptors [51]. Intriguingly, among the four potential ligands of SjGIPC3 identified in this study, one is the glutamate receptor NMDA, which also bears a class I motif “TTTL”; thus, we cannot rule out the possibility that SjGIPC3 may also have a physiological function in the regulation of NMDA receptor trafficking, similar to its orthologue in mouse.

## Conclusions

For the first time, we have presented the molecular characterization of the PDZ domain-containing protein GIPC3 from *S. japonicum*. We defined the ligand binding specificity of the SjGIPC3-PDZ domain by screening a random peptide library constructed with human genomic DNA. The ligand binding ability of the SjGIPC3-PDZ domain is relatively extensive; it can bind both type I and type II ligand motifs, which hints at its potential to play multiple roles during the development of *S. japonicum*, especially in the adult stage. Our study also provided a practical method for predicting potential ligands of SjGIPC3. Ongoing studies to identify more ligands involved in signal transduction followed by improvement of the quality of the *S. japonicum* proteomic dataset will assist in the discovery of novel drug targets against the parasite.

### Ethics statement

All procedures performed on animals within this study were conducted following animal husbandry guidelines of the Chinese Academy of Medical Sciences and with permission from the Experimental Animal Committee.

## Competing interests

The authors declare that they have no competing interests.

## Authors’ contributions

YM, PC, and YG conceived and designed the experiments. YM, HH, SL, and PC performed the experiments. YM, PC, and YG analyzed the data. YM, PC, and YG wrote the manuscript. All authors read and approved the final version of the manuscript.

## Supplementary Material

Additional file 1 **Table S1. **Primer sequences used in this study. This file contains the information about the primers used for qRT-PCR and molecular cloning. Click here for file
